# Insulin/carbohydrates ratio during the first 6‐month therapy with insulin degludec in a paediatric population with type 1 diabetes previously treated with insulin glargine. An observational longitudinal study

**DOI:** 10.1002/edm2.121

**Published:** 2020-03-12

**Authors:** Stefano Tumini, Olimpia Iacono, Laura Comegna, Elisabetta Fioretti, Paola Guidone, Gabriella Levantini, Daniele Panichi, Milena Catenaro, Ilaria Rossi, Flavia Amaro, Giusi Graziano, Maria Chiara Rossi, Paola Cipriano

**Affiliations:** ^1^ Department of Pediatrics University of Chieti Chieti Italy; ^2^ CORESEARCH – Center for Outcomes Research and Clinical Epidemiology Pescara Italy

**Keywords:** carbohydrates, continuous subcutaneous insulin infusion, degludec, glargine, insulin analogues, paediatric diabetes, type 1 diabetes

## Abstract

**Background:**

No data exist about the changes induced by the transition from first‐generation long‐acting insulins to second‐generation long‐acting analogues in the paediatric population.

**Objective:**

To assess changes in insulin/carbohydrate ratio (I:CHO) after the first 6 months of degludec therapy in a paediatric population with type 1 diabetes previously treated with glargine U100.

**Subjects:**

All patients treated with degludec under routine clinical practice conditions were retrospectively analysed.

**Methods:**

Nonprofit observational retrospective study. Changes during the follow‐up in mean CHO/I ratio were assessed using longitudinal linear models for repeated measures. Rate of hypoglycaemia, ketoacidosis and adverse events was evaluated.

**Results:**

Overall, 51 children (mean age 13.8 ± 4.6 years; mean diabetes duration 5.8 ± 3.9 years) started therapy with degludec in the period between April 2017 and April 2018. I:CHO ratio before starting degludec therapy significantly differed among the three meals, being the lowest at breakfast and the highest at dinner. After introducing degludec, I:CHO ratio at lunch (−1.29 95% CI −2.02;−0.57) and at dinner (−3.08 95% CI −4.35;−1.8) significantly decreased, while it slightly increased at breakfast (+1.37 95% CI 0.47;2.28). No episodes of severe hypoglycaemia, ketoacidosis and adverse event were recorded during 6 months.

**Conclusions:**

Our data show that the use of degludec is associated with a significant change in the I:CHO ratio at the different meals compared to the previous glargine therapy. This could derive from the flat and prolonged pharmacokinetic profile of degludec. This has important clinical implications for daily insulin dose adjustments.

## Graphical abstract

No data exist about the changes induced by the transition from first‐generation long‐acting insulins to second‐generation long‐acting analogues in the paediatric population. The study assessed changes in insulin/carbohydrate ratio (I:CHO) after the first 6 months of degludec therapy in a paediatric population with type 1 diabetes previously treated with glargine U100. Our data showed that the use of degludec is associated with a flattening in the I:CHO ratio at the different meals compared to the previous glargine therapy.

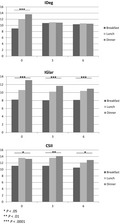

## 1 INTRODUCTION

In Italy, about 15 000 children and adolescents suffer from type 1 diabetes (T1DM).^1^ Recent epidemiological data suggest an increase in incidence in class 0‐4 years, which prolongs the years of exposure to hyperglycaemia,^2^ with an increased risk of complications in adolescence and adulthood.^3^, ^4^, ^5^ Insulin therapy and glycaemic control are essential for preventing acute and long‐term complications and overall well‐being in people with T1DM.^6^ The recommended therapy scheme is basal‐bolus, which involves the use of short‐acting and long‐acting insulin analogues.^7^ Currently, in Italy three types of short‐acting insulin analogues are available (Lispro, Aspart, Glulisine) and four basal insulin analogues (Glargine, Detemir, Insulin lispro protamine and Degludec). In addition, basal insulinization is achieved with the use of the short‐acting analogue (Lispro, Aspart, Glulisine) in patients on continuous subcutaneous insulin infusion (CSII).^7^

Degludec (IDeg) is a basal analogue of insulin which differs from the other basal analogues for the duration of action of more than 24 hours (with a half‐life of 25 hours up to 42 hours) and for a metabolic effect evenly distributed throughout the day. In phase III studies, IDeg stable profile was proved to be particularly protective in terms of nocturnal hypoglycaemia; furthermore, it allowed flexibility in time of administration and this was associated with better Quality of Life (QoL).^8^, ^9^, ^10^ IDeg has been the first analogue approved for the clinical use in paediatric population, starting from the first year of age.

The estimation of the meal carbohydrate content is considered nowadays the best method for calculating the preprandial dose of short‐acting insulin.^11^, ^12^ The dose of preprandial insulin is calculated by the patients, which are educated to follow a flexible algorithm based on the carbohydrate content of the meal, the insulin/carbohydrate ratio (I:CHO) and the insulin sensitivity factor (ISF). The ISF allows the correction of any preprandial hyperglycaemia.^13^, ^14^

The use of analogues with a half‐life of 25 hours and a kinetics elimination of first degree allowed to obtain a basal insulinization profile homogeneously distributed during the day. No attenuation at the steady state (generally after a time interval equal to three times the half‐life) of the hypoglycaemic effect at the end of 24 hours was documented. A long half‐life allows a stable basal profile and appears to be protective in case of a missed basal insulin dose or administered twice by mistake.^15^

It is therefore evident that a constant preprandial insulinization determines substantial changes in the calculation of the short‐acting preprandial dose, especially for subjects who adequately modify the doses on the basis of the CHO contribution. However, there are no data in the literature about the changes induced by the transition from first‐generation long‐acting insulins with a half‐life of 12 hours to second‐generation long‐acting analogues with a half‐life of 25 hours.^16^

The present study aimed to assess changes in I:CHO ratio and insulin sensitivity factor (ISF) during the first 6 months of IDeg therapy in a paediatric population with T1DM previously treated with glargine U100 (IGlar). For descriptive purposes, children treated with other insulin schemes (ie, multiple daily injections with insulin glargine as basal insulin or CSII) were also considered.

## 2 MATERIALS AND METHODS

This was a nonprofit longitudinal retrospective observational study conducted in a regional paediatric diabetes clinic (Chieti, Italy). All patients with type 1 diabetes treated with IDeg under routine clinical practice conditions and with at least 6‐month follow‐up were considered.

Exclusion criteria were as follows: other diabetes type, age <1 year or ≥18 years, recent diabetes diagnosis (<1 year), use of systemic glucocorticoids (except for formulations for topical and inhalation application) for seven or more consecutive days during the last 3 months, known hypothyroidism or hyperthyroidism not adequately controlled, poor adherence to the gluten‐free diet in case of coeliac disease, treatment with oral glucose‐lowering drugs at any time after diagnosis.

Information on gender, age, diabetes duration, weight, body mass index (BMI), pubertal stage (Tanner classification), HbA1c, diabetes therapy, severe hypoglycaemia and ketoacidosis was extracted by the electronic clinical record system adopted in the clinic.

Data on mean blood glucose were obtained from the download of patient glucose metres.

No patient used flash glucose monitoring or continuous glucose monitoring since these devices were not reimbursed by Italian National Heath System while the study was conducted.

Baseline (ie, date of the first prescription of the insulin scheme) variables included age, gender, duration of diabetes, body weight, body mass index (BMI), insulin regimen, HbA1c and mean blood glucose levels in the previous 3 months.

Study end‐points included changes during the follow‐up in mean CHO/I ratio (primary end‐point) and ISF.

Based on clinical recommendations, patients attended a visit approximately every 3 months.

All patients were managed according to standard care. Specifically, in the centre, carbohydrate counting and education on glycaemic index or insulin coverage of high‐fat, high‐protein meals are commonly introduced at onset of disease.^17^

CHO are quantified on the basis of gram increment. At onset of disease, most prepubertal patients require 1 extra unit of insulin for each 10‐15 g of carbohydrate ingested, pubertal patients can require up to 1 unit for 6 g CHO increment; infants and toddler require 1U each 30‐60 g.

Similarly, ISF is typically 1U: 200‐270 mg/dL in infant/toddler; 1U: 100‐180 mg/dL in prepuberty; 1U: 90‐100 mg/dL in early puberty and 1U: 35‐50 mg/dL in adolescence.

Blood glucose self‐monitoring (pre‐ and postprandial) provides information on postprandial glucose excursions and the need to modify I:CHO ratio, adjust the prandial insulin timing or amount, or alter the insulin delivery or dose for meals high in fat and protein (typically pizza).

Carbohydrate counting accuracy is verified during routine visits and, when needed, by phone contact.

Insulin/carbohydrate ratio and ISF per type of meal are adjusted, if needed, at the discretion of the patient/parent/clinician when blood glucose levels are persistently (last 4‐7 days in the absence of interfering events, for example unplanned physical activity or intercurrent illness) over the target (preprandial plasma glucose: 71‐145 mg/dL for breakfast and lunch and 120‐180 mg/dL for bedtime).

Optimal titration of I:CHO ratio is successively determined individually on the basis of personal food preferences and individual physiological response.

ISPAD 2018 guidelines were followed for insulin dose adjustments during physical activity and intercurrent illness and for the management of weight increase.^18^, ^19^ Weight change during the study was evaluated as standardized BMI (BMI SDS) according to the Italian Standards.^20^

This research was conducted in accordance with the guidelines of the Declaration of Helsinki. The study was approved by the local ethics committee, and informed consent was obtained from all participants.

### 2.1 Statistical analysis

An exploratory analysis conducted in our centre on 50 children documented a I:CHO of 10 ± 3. A sample size of 44 subjects achieves 90% power to detect a mean of paired differences of 1.5 with an estimated standard deviation of 3.0 and with a significance level (alpha) of 0.05 using a two‐sided paired *t* test.

Baseline characteristics were expressed as mean and standard deviation or percentage for continuous and categorical variables, respectively.

Longitudinal linear models for repeated measures were applied to assess trends over time in continuous end‐points. All longitudinal models took into consideration three time points, that is date of switch (T0), 3‐month follow‐up (T + 3) and 6‐month follow‐up (T + 6). An unstructured correlation type was used to account for within‐patient correlation over time. Results were expressed as estimated mean and estimated mean change from baseline with their 95% confidence intervals (CIs). *P*‐values <.05 were considered statistically significant.

Number and percentage of patients with at least one a severe hypoglycaemia or ketoacidosis episode were assessed.

Given the substantial differences in patients' characteristics, no formal comparison between the different insulin schemes was performed.

All analyses were performed using sas software release 9.4 (SAS Institute).

## 3 RESULTS

Overall, 51 children started therapy with IDeg in the period between April 2017 and April 2018. Additional information on the other two cohorts available in the clinical database of the centre included 27 patients treated with IGlar and 23 treated with CSII. Baseline characteristics by basal insulin regimen are reported in Table 1. All patients administered IDeg at lunch time and used glucose metres for the daily measurements of blood glucose.

**Table 1:** Baseline characteristics

	IDeg	IGlar	CSII	*P*‐value*
N	51	27	23	
Males (%)	60.8	55.6	30.4	**.05**
Age (y)	13.8 ± 4.6	14.1 ± 3.3	13.0 ± 4.2	.49
Diabetes duration (%)	5.8 ± 3.9	5.3 ± 3.4	8.5 ± 3.9	**.008**
Weight (kg)	52.8 ± 19.3	57.7 ± 16.7	51.1 ± 17.8	.35
BMI (kg/m^2^)	21.1 ± 4.2	21.4 ± 3.2	21.6 ± 3.8	.70
Puberal stage (%)				
Prepubertal	29.4	11.1	34.8	.11
Pubertal	70.6	88.9	65.2	
HbA1c (%)	7.6 ± 0.9	7.2 ± 0.8	7.6 ± 0.7	.08
HbA1c (mmol/mol)	59.1 ± 10.4	54.8 ± 8.3	59.4 ± 7.9	.08
Mean daily blood glucose in the previous 3 mo (mg/dl)	166.5 ± 31.8	156.4 ± 25.3	165.7 ± 20.6	.17

* Statistically significant (*P*‐value <.05) changes vs. T0 values are in bold text.

**Figure 1:**
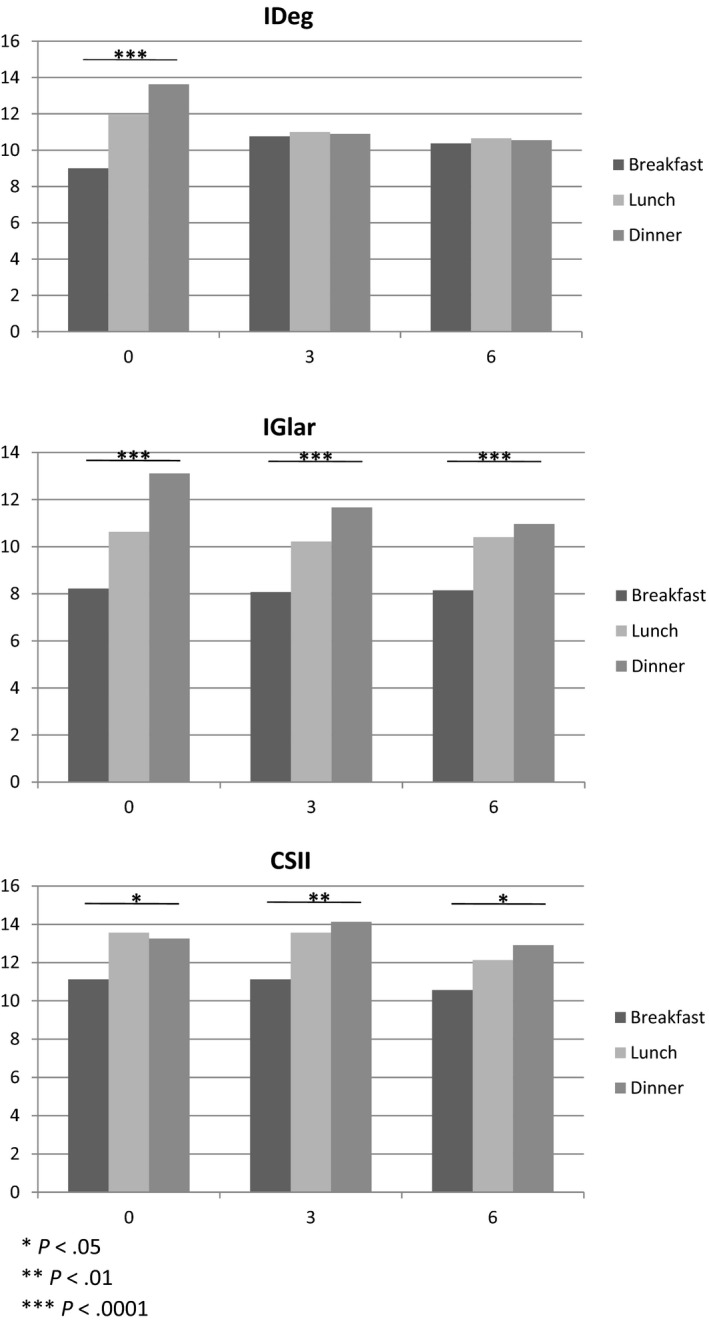
Changes during 6 mo in CHO/I ratio by meal

Figure 1 shows changes in I:CHO ratio during the follow‐up in the IDeg group. Additionally, trends obtained in the other two groups are shown. In the IDeg group, I:CHO ratio before starting IDeg therapy significantly differed among the three meals, being the lowest at breakfast and the highest at dinner. After introducing IDeg, I:CHO ratio at lunch and at dinner significantly decreased, while it slightly increased at breakfast (Table 2). As a result, the difference in I:CHO ratio among meals disappeared.

**Table 2:** Trends over time in continuous end‐points in the IDeg group: results of mixed linear models

Outcome	Time‐point	Estimated mean values (95% CI)	Estimated mean change from T0 (95% CI)	*P*‐value*
CHO/I ratio (breakfast)	T0	9.00 (7.89‐10.11)	–	–
	T + 3	10.76 (9.64‐11.89)	+1.76 (0.97;2.56)	**<.0001**
	T + 6	10.37 (9.22‐11.52)	+1.37 (0.47;2.28)	**<.05**
CHO/I ratio (lunch)	T0	11.94 (10.54‐13.35)	–	–
	T + 3	11.00 (9.83‐12.17)	−0.94 (−1.66;−0.22)	**.01**
	T + 6	10.65 (9.49‐11.81)	−1.29 (−2.02;−0.57)	**<.001**
CHO/I ratio (dinner)	T0	13.63 (11.75‐15.51)	–	–
	T + 3	10.90 (9.8‐12.01)	−2.73 (−3.98;−1.47)	**<.0001**
	T + 6	10.55 (9.4‐11.7)	−3.08 (−4.35;−1.8)	**<.0001**
Insulin sensitivity factor	T0	73.92 (61.15‐86.69)	–	–
	T + 3	67.65 (56.94‐78.35)	−6.27 (−12.09;−0.46)	**.03**
	T + 6	65.00 (55.56‐74.44)	−8.92 (−15.8;−2.05)	**.01**

* Statistically significant (*P*‐value <.05) changes vs. T0 values are in bold text.

In the other insulin schemes, no major changes were documented during the follow‐up, and I:CHO ratio remained higher at dinner as compared to breakfast.

**Figure 2:**
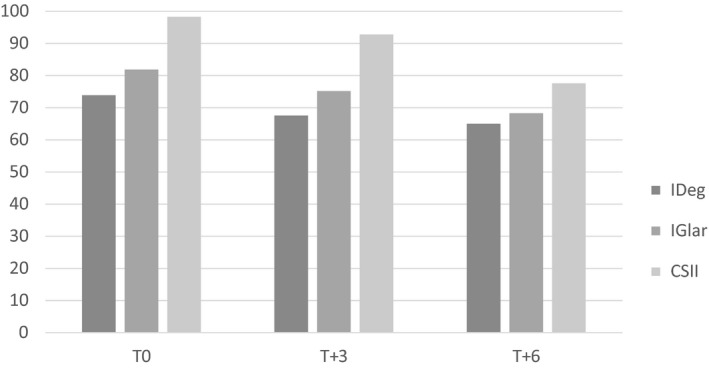
ISF by treatment group and time

Figure 2 shows changes in ISF during the follow‐up. ISF significantly decreased after 3 and 6 months from the start of IDeg (Table 2).

During the study, no change in BMI SDS was registered in the three groups.

No episodes of severe hypoglycaemia, ketoacidosis or other adverse events occurred during the observation period.

## 4 DISCUSSION

Our study shows that, after initiation of IDeg, I: CHO ratios at lunch and dinner decrease, becoming similar to the breakfast value. The other two therapeutic subgroups could not be compared with IDeg since patients' characteristics were systematically different. However, descriptive data showed the persistence of different values in correspondence of the three main meals.

In addition, longitudinal analyses performed on IDeg group showed that the ‘flattening’ of I:CHO derives from a slight increase in I:CHO at breakfast and a marked reduction in I:CHO at lunch and especially at dinner.

This effect on I:CHO ratio is presumably due to a greater pharmacokinetic and pharmacodynamic stability of IDeg as compared to the other basalization strategies.

As for the secondary end‐points assessed in the IDeg group, ISF was significantly reduced from baseline to 6 months after IDeg initiation. Different explanations could be hypothesized: the educational reinforcement during visits due to the prescription of a new basal insulin, the physiological increase in weight in paediatric age, especially in the adolescent age, and a greater predictability of the response to preprandial insulin boluses linked to the use of a flat second‐generation basal analogue. The lack of differences in the ISF between breakfast, lunch and dinner was an expected result.

For mere descriptive purposes, we documented that ISF levels even in the other two treatment groups declined during the follow‐up, but they remained higher than in the IDeg group.

Furthermore, no episodes of severe hypoglycaemia, ketoacidosis and adverse event were recorded.

The study has important implications for clinical practice. Many patients and many parents tend to increase the basal insulin dose in order to limit the possible hyperglycaemic effect of the snacks, to avoid an extra insulin bolus or because they are afraid of the increase of the rapid analogue dose. In this regard, many patients and families are not able to manage hypoglycaemia, to control premeal insulin doses and to perform the CHO counting.^21^ As a result, they tend to administer an insufficient rapid analogue dose with a consequent postprandial hyperglycaemia. Furthermore, the excessive basal insulin dose can cause the necessity of CHO intake to avoid hypoglycaemia, with a consequent hyperglycaemia.^21^

Strich et al^21^ documented that well managed patients need a basal insulin dose of 0.28 ± 0.08 U/Kg/die (35 ± 10% of the daily total dose) and they suppose that the main problem of these patients is their inability to manage the insulin bolus.

It could be supposed that beginning to use the basal analogues of third generation in patients who are not able to use the algorithm based on the CHO intake and the starting blood glucose level cannot improve the metabolic control or may even worsen it. Currently, there are a few studies on the use of the third‐generation basal analogues in paediatric age, all concerning IDeg, and often including also adult patients.^22^, ^23^, ^24^, ^25^

The study has strengths and limitations. As a main strength, this is the first study focusing on the impact of IDeg on I:CHO and ISF in a paediatric population, under routine clinical practice conditions. As a limitation, due to the study design, no formal between‐group statistical comparisons were allowed; however, the within group pre‐post comparison in the IDeg group was the main focus of the analysis.

## 5 CONCLUSIONS

Our data show that the use of IDeg is associated with a significant change in the I:CHO ratio at the different meals compared to the previous IGlarg therapy. This could derive from the flat and prolonged pharmacokinetic profile of IDeg. This can represent an advantage, since it makes the calculation of the preprandial bolus easier. Patients must therefore be instructed and education reinforced on CHO counting; they should be advised that doses of preprandial insulin may need significant changes after switching to IDeg therapy, in order to obtain more appropriate preprandial insulin doses.

## CONFLICT OF INTEREST

ST has participated in advisory boards for Novo Nordisk, Sanofi Aventis, Eli Lilly and Lifescan. He has also received speaker honoraria from Roche Diagnostics, Novo Nordisk, Sanofi Aventis and Harmonium Pharma. MCR received funding for research from Sanofi, Novo Nordisk, Theras, Alfasigma. The remaining authors have no disclosure.

## AUTHORS' CONTRIBUTION

S.T. conception and design, data interpretation, drafting the manuscript, guarantor of accuracy and integrity of the manuscript. O.I., L.C., E.F., P.G., G.L., D.P., M.C., I.R., F.A. and P.C. acquisition of data, critical revision and approval of accuracy and integrity of the manuscript. G.G. data analysis. M.C.R. data analysis and drafting the manuscript.

## Data Availability

The data that support the findings of this study are available from the corresponding author upon reasonable request.
